# Supplementary material for: Metoprolol rescues endothelial progenitor cell dysfunction in diabetes
Source: PeerJ. 2020 Jul 7;8:e9306. doi: 10.7717/peerj.9306 (PMC7350924; doi:10.7717/peerj.9306)

**Metoprolol** **Rescues Endothelial Progenitor Cell Dysfunction in Diabetes**

Lang Yan^1, †^, Yi-fan Dong^1, †^, Ya-ping Deng^2^, Xue Han^2^, Wen-jing Shi^1^, Jing-feng Li^1^, Fang-yuan Gao^1^, Xiao-fang Zhang^1^, Yi-jun Tian^1^, Xiao-yu Dai^1^, Jiang-bo Zhu^1^, Ji-kuai Chen^1^

**Affiliations**

^1^ Department of Health Toxicology, Faculty of Naval Medicine, Second Military Medical University, Shanghai 200433, China.

2. Department of Pharmacy, Zhejiang Xiaoshan Hospital, Hangzhou, Zhejiang, China

**Corresponding Author:**

Ji-kuai Chen, Department of Health Toxicology, Second Military Medical University, 800 Xiangyin Road, Shanghai, 200433, China. E-mail: cjk.smmu@hotmail.com

Or Jiang-bo Zhu, Department of Health Toxicology, Second Military Medical University, 800 Xiangyin Road, Shanghai, 200433, China. E-mail: jiangbozhu1@163.com.

^†^ These two authors contributed equally to this work.

**Figure legends**

**Figure S1** Changes of blood glucose concentration and body weight of streptozotocin-induced diabetic mice (STZ). Blood glucose (A) and body weight (B) changes of STZ (60 mg/kg 5d, i.p.) treated mice, which was defined as diabetic mice (blood glucose value > 13.8 mmol/L) 15 days after STZ treatment. Values are mean SEM. n=6 per group. **p* < 0.05 vs. Control.

Figure S1


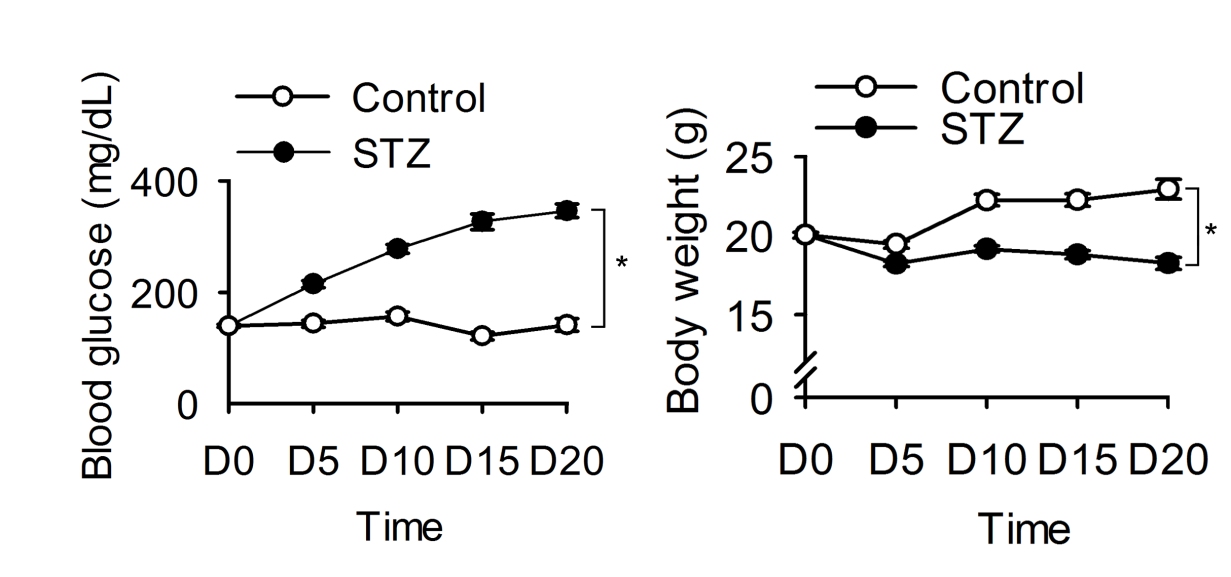

Supplement: Supplemental Information 1 — Blood glucose (A) and body weight (B) changes of STZ (60 mg/kg 5d, i.p.) treated mice, which was defined as diabetic mice (blood glucose value > 13.8 mmol/L) 15 days after STZ treatment. Values are mean SEM. n = 6 per group. * p < 0.05 vs. Control. [file peerj-08-9306-s001.docx]
